# Bioprospecting of Essential Oil-Bearing Plants: Rapid Screening of Volatile Organic Compounds Using Headspace Bubble-in-Drop Single-Drop Microextraction for Gas Chromatography Analysis

**DOI:** 10.3390/plants11202749

**Published:** 2022-10-17

**Authors:** Thabiso E. Letseka, Ntjana J. Sepheka, Ian A. Dubery, Mosotho J. George

**Affiliations:** 1 Department of Chemistry and Chemical Technology, National University of Lesotho, P.O. Box 180, Roma 100, Lesotho; 2 Department of Biochemistry, University of Johannesburg, P.O. Box 524, Johannesburg 2006, South Africa

**Keywords:** bubble-in-drop, essential oils, headspace, oil-bearing plants, SDME, volatiles

## Abstract

Essential oils are vital constituents of oil-bearing plants. However, their screening still demands harvesting of the plant for laboratory analysis. We report herein a simple, rapid and robust headspace bubble-in-drop microextraction screening technique (BID-SPME) requiring only small amounts of plant material. The optimised method uses 0.5 g of the crushed plant leaves sample obtained in a 2 mL capped chromatography vial, heated to 55 °C and sampled with 2 µL heptadecane in a Hamilton gastight syringe equilibrated for 15 min exposed to the headspace volume. The method was applied to three plants, *Pinus radiata*, *Tagetes minuta* and *Artemisia afra*, which are known for their essential oil content. The method was able to extract at least 80% of the oil constituents in such abundance that they could be easily annotated using the gas chromatography–mass spectrometry (GC–MS) mass spectral libraries. The major volatile organic compounds (VOCs) detected included tagetone, terpinen-4-ol, ocimenone, caryophyllene, dihydrotagetone, terpinolene and artemisia ketone, just to mention a few, at different concentrations in different plants. Importantly, these annotated VOCs were also reported in other studies in the same and even different plants, extracted using normal steam distillation and importantly those reported in the literature for different extraction techniques.

## 1. Introduction

Essential oils associated with aromatic plants play quite a number of roles such as acting as chemical deterrents and providing defense especially against herbivorous insect pests and pathogens [1]. Due to their strong scent, these aromatics assist in plant–plant interactions and help to attract insects for pollination [2]. Pharmaceutically, medicinal oil-bearing plants are used for various ailments due to their potential anti-oxidant activity, anti-inflammation, anti-microbial, anti-aging and other medical properties [3,4,5,6].

Essential oils, also known as volatile oils or ethereal oils, are aromatic oily liquids obtained from various aromatic plant parts. They are not produced by all plants but, rather, their occurrence is restricted to over 200 plant species from about 60 families. These plant families are the Asteraceae, Myrtaceae, Pinaceae, Zingiberaceae, Umbelliferae, Lamiaceae, Apiaceae, Rutaceae, and Poaceae [7]. There are about 3000 essential oils known, but only 300 are found to be economically important, and they find most applications in flavour and fragrance industries. Essential oils are a complex mixture of mainly terpenes; particularly monoterpenes and sesquiterpenes and their oxygenated derivatives such as alcohols, aldehydes, esters, ethers, ketones, phenols and oxides [8]. Essential oils are formed in the cytoplasm of secretory cells and are isolated from whole plant or plant parts such as flowers, buds, seeds, leaves, fruits and roots.

Essential oils represent one of the most important components in oil-bearing plants that are used for their aromatic value and are thus in high demand by the food and beverage sectors, the pharmaceutical industry and as industrial products. Only some essential oils are available commercially as natural product-based additives [9]. Due to high demand from these sectors, together with low oil yields, massive amounts of plant material are required, leading to potential over-harvesting [10,11,12]. For extraction, several methods that include steam distillation, solvent extraction, maceration and hydro-distillation are used. Steam distillation is the most widely used method due to its affordability and simplicity [10,12], while also preserving the properties of the oils after the extraction [6,10]. Other methods such as supercritical carbon dioxide that also preserve the nature of the oils are quite expensive and more complex to be employed routinely in laboratories with low budgets.

Due to their volatile nature, it is important to study the chemical composition of the volatile fraction once the essential oil is extracted [6,10]. The headspace (HS) sampling of plant volatiles has generated attention due to its suitability for automation [13] as well as being environmentally friendly and easy to implement. The HS is described as the gas phase (in equilibrium or not with the matrix) above a solid or liquid sample, when this is placed in a chromatographic vial sealed with a septum [14]. Static HS sampling [15,16] involves the heating of a system for a period of time at a set temperature and allowing the distribution of analytes between the sample and the gas phase which then will allow for the retraction of a fraction of the HS for gas chromatography (GC) analysis. This method is fairly simple and does not require complicated instrumentation. However, sensitivity is limited, as it can only allow detection of the most intense and abundant odorants [15]. On the other hand, dynamic HS sampling [13], also known as purge-and-trap, is a non-equilibrium, continuous gas extraction technique. This involves a flow of inert gas for the continuous extraction of volatile compounds from a sample and their further pre-concentration onto an adsorbent or cryogenic trap before the GC analysis. Although the dynamic method is technically more complicated to perform than its static counterpart, it does provide a higher yield of the VOCs.

While these extraction methods are effective, they require wasteful harvesting of considerable amounts of plant material. Consequently, there is a need for rapid and less consumptive methods that can be used for screening or profiling. Volatile organic compounds (VOCs) are the simplest to screen. However, there are still relatively few reports where field screening is carried out without the need to harvest the plant. Among the mostly used methods for HS sampling with minimal sample volume is the solid-phase microextraction (SPME) technique [16]. Despite the advances in the chemistry and other properties of the adsorbent fibres used in SPME [17], this technique is still considered expensive and time consuming. In addition, the fibres are fragile and sometimes too selective depending on the material used, making them less competitive compared to the liquid-based counterparts that include the hollow fibre membranes impregnated with organic solvents [18] and drop-based formats [19]. However, these techniques are not effective for field work due to the volatility of organic solvents.

Herein, we report the development and application of an HS bubble-in-drop single drop microextraction (BID-SDME) method which involves a droplet of organic solvent with an air-bubble engulfed inside the droplet [20] for the sampling of plant VOCs. The BID-SDME method has been reported to increase the extraction kinetics of analytes through an increased surface area to volume ratio, thus contributing to a concentration effect. This method has been reported primarily for the direct immersion mode for analyses of different compounds in varying matrices that include plant, animal and environmental health [21,22,23,24,25,26]. It is noteworthy that so far, only a single report is found that describes the use of HS sampling of some VOCs using the BID-SDME method [27].

## 2. Results and Discussion

### 2.1. Optimisation of Screening Technique for the Essential Oils

#### 2.1.1. Selection of the Extracting Solvent

**Figure 1:**
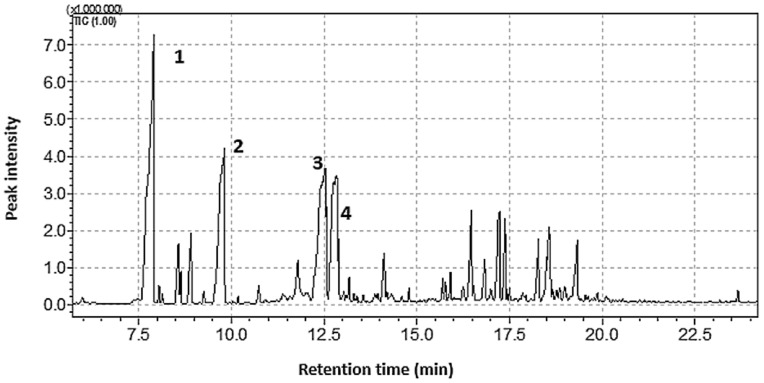
A chromatogram of the 20-min headspace single-drop micro-extraction (SDME) extract of the commercial oil with 2 µL heptadecane and analysed using gas chromatography with flame ionisation detector.

The first task was to select the solvent to be used for extraction, as the solvent has to either be more volatile than the VOCs so that it will elute before the VOCs or be less volatile so that it would elute later than the VOCs. However, the challenge with more volatile solvents is that they evaporate during the extraction; hence, they are not suited for HS sampling. Accordingly, two solvents, dodecane ((CH_3_–(CH_2_)_10_–CH_3_), boiling point = 216 °C) and heptadecane ((CH_3_–(CH_2_)_15_–CH_3_), boiling point = 302 °C), were tested for this purpose. Of the two, dodecane co-eluted with some of the volatiles, thus making heptadecane a solvent of choice given its later retention time and a higher temperature of elution. Figure 1 shows the extract of the GC-FID chromatogram obtained when 0.5 mL of the commercial pine oil was sampled with 2 µL of heptadecane for 20 min. Since the compounds could not be identified by GC–FID, the four most dominant compounds labelled 1–4 in Figure 1 were used for further optimisation.

#### 2.1.2. Optimisation of Extracting Solvent (Heptadecane) Volume

**Figure 2:**
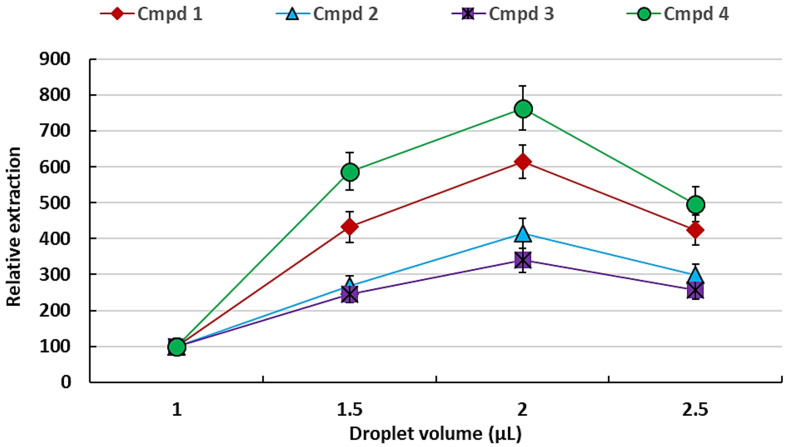
Effect of heptadecane droplet volume (relative to 1 µL) on the extraction of VOCs following a 10 min equilibration time. The error bars indicate the standard deviation for *n* = 3 replicates.

After the determination of the extracting solvent, the determination of the optimum volume of the solvent (to effectively concentrate the analytes and that would be stable when suspended from the syringe) was investigated. A series of droplet volumes ranging from 1 to 2.5 μL were tested to determine the extraction with varying solvent volume (Figure 2) using 10 min extraction time as a starting extraction time without optimisation.

From Figure 2, it can be seen that the extraction efficiency (as reflected by peak intensity) increases with solvent volume and reaches a maximum at 2 μL and then dropped at 2.5 μL. The drop in the efficiency at 2.5 μL may be due to a dilution effect of analytes on account of the increased volume of solvent. This observation correlates with the original report on BID-SDME where it was reported that the increase in surface area is more than the volumetric increase with increasing drop size [22]. It was further observed that a larger drop size led to drop instability and the drop falling off the syringe tip. Therefore, the volume of 2 µL was chosen as the optimum volume of the droplet and used for further experiments.

#### 2.1.3. Optimisation of Extraction Temperature

Extraction temperature plays an important role in the determination of the efficiency of the extraction, since it increases the vapour pressure of the volatile compounds, hence enriching them in the HS [15]. The temperature effect was studied from 25 to 55 °C by extracting the samples incubated at different temperatures using a water bath.

**Figure 3:**
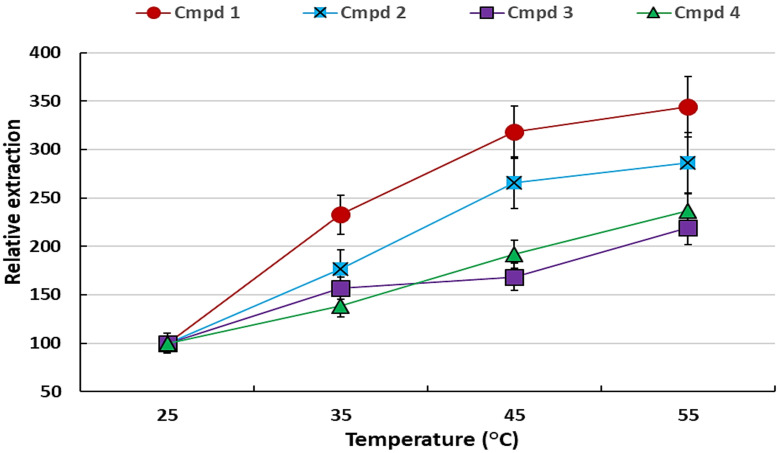
The effect of temperature on the headspace SDME of the VOCs using 2 µL heptadecane and 10 min equilibration relative to 25 °C. The error bars indicate the standard deviation for *n* = 3 replicates.

Figure 3 shows that an increase in temperature resulted in the increase in the extraction of the VOCs between 2 and 3-fold (200–300%) relative to room temperature (25 °C). However, this increase could not be studied at temperatures higher than 55 °C because the organic solvent drop became unstable at higher temperatures. As a result, 55 °C was selected to be the optimum extraction temperature and thus used for further experiments.

#### 2.1.4. Optimisation of the Extraction Time

**Figure 4:**
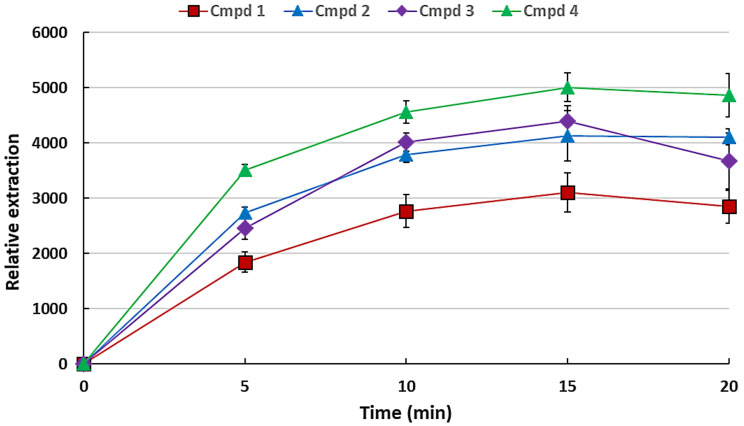
Variation of extraction time on the headspace SDME of the VOCs using 2 µL heptadecane incubated at 55 °C. The error bars indicate the standard deviation for *n* = 3 replicates.

It is essential to determine the optimum extraction time for sampling of the VOCs since this method is equilibrium based rather than exhaustive, and this makes it time-dependent. The study was conducted in the time interval of 5–20 min to determine the optimum extraction time. Figure 4 shows the variation of extraction with varying extraction time periods.

As can be seen from Figure 4, the extraction efficiency increases with extraction time and levels off at about 15 min for the standards used. This levelling off could be attributable to the equilibration of the VOCs between the HS and the organic droplet, as the process is not exhaustive. Therefore, 15 min equilibration was chosen as the optimum extraction time and was consequently used for further experiments.

#### 2.1.5. Optimisation of the Sample Volume

The amount of sample in the sampling container (e.g., a chromatographic vial) is important as it reduces the HS volume, thus increasing the net concentration of the VOCs in the HS. This is in addition to the obvious increase in the VOCs consequent to the increased amount of sample. To vary the HS volume, varying volumes of sample from 25 to 1500 μL were added to different vials and sampled accordingly for 15 min at 55 °C.

**Figure 5:**
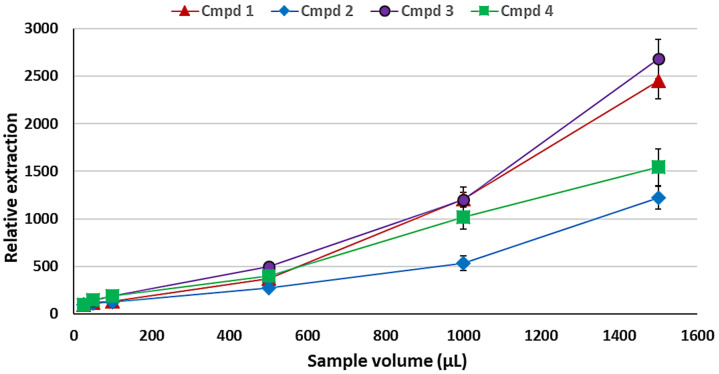
Effect of varying sample volumes (25–1500 µL) on the SDME using 2 µL of heptadecane with 15 min equilibration at 55 °C. The error bars indicate the standard deviation for *n* = 3 replicates.

It is seen in Figure 5 that the increase in sample volume leads to concomitant increases in VOC extraction. However, this increase is not linear, perhaps since this increase does not only increase the amount of the VOCs in the HS but also reduces the HS volume, thus effectively concentrating the analytes. Given that the development of the method is aimed at qualitative analyses, the sample volume of 1.5 mL (1500 µL) was used as an optimum volume of the sample.

### 2.2. Comparison of SDME to BID-SDME on the Headspace Extraction of the VOCs

Since the SDME method is limited to drop surface area, a variation of the method was introduced to increase the surface area of the drop, known as the BID-SDME, with the aim of increasing the extraction efficiency and kinetics. The optimised conditions as described above were used in the comparison and the volume of the air bubble and droplet were taken from literature values. Using 2 μL drops while preserving the drop to bubble ratio of 2:1 (a 2 μL droplet to 1 μL air bubble) that was determined elsewhere [26] failed as BID-SDME assembly became relatively unstable, resulting in the loss of the droplet. The volumes were then scaled down by half, resulting in a 1 μL drop and 0.5 μL bubble, which were then compared against a 1 μL drop for SDME.

**Figure 6:**
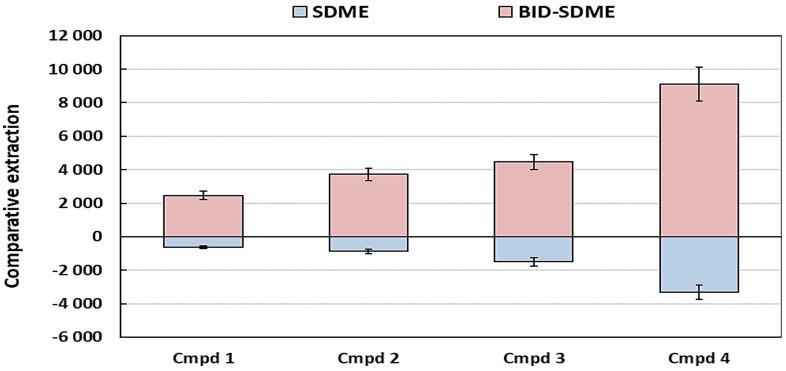
Back-to-back comparison of the SDME and BID-SDME techniques using 2 µL heptadecane with 15-min equilibration at 55 °C and a sample volume of 1.5 mL. The bars indicate the magnitude of the relative peak areas, while the error bars represent the standard deviations of the triplicate analyses.

It is clearly seen in Figure 6 that the BID-SDME technique offers higher extraction efficiency than the classical SDME technique due to the increase in droplet surface area. The 2:1 drop to bubble ratio was further used for BID-SDME combined with the optimised parameters for application in real plant samples.

### 2.3. Application of the Developed Method to Real Samples

Following the optimisation of the method, plant leaves were obtained from three oil-bearing plants that are used for different purposes locally. The leaf materials were from pine trees, locally known as phaena (*Pinus radiata* D. Don, Clade: Gymnosperms), wild marigold or ‘kakiebos’, known locally as monkhane, nkhi or lechuchutha (*Tagetes minuta* L., Clade: Angiosperms, Asterales) and African wormwood, known locally as lengana (*Artemisia afra* Jacq. ex Willd, Clade: Angiosperms, Asterales). These were coarsely ground using pestle and mortar and thereafter transferred into 2 mL GC vials and capped accordingly. It must be noted that there was no optimisation of the plant amount as opposed to the aqueous solution used in the preliminary experiments. This is because it was observed that the mass of 1 g was already too high in the vial, and increasing to 1.5 g—corresponding to the 1.5 mL of the aqueous oil suspension—would fill the vial and leave no space for the droplet. Higher amounts of plant material could also lead to some leaf fragments touching the droplet and leading to drop loss.

**Figure 7:**
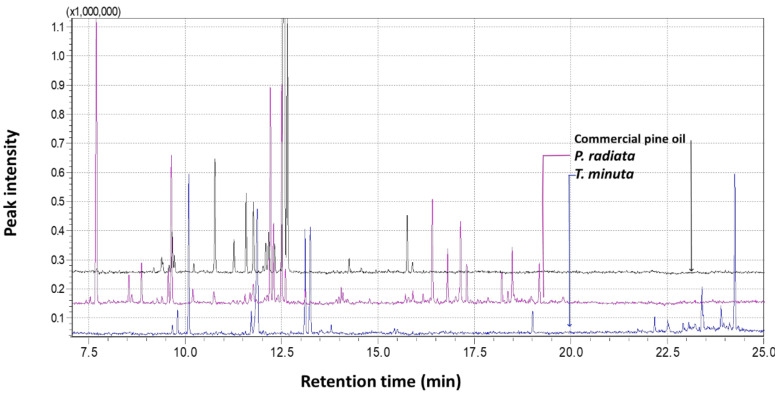
Representative chromatograms of the GC-MS analysis of the volatiles from *T. minuta* (wild marigold) and *P. radiata* (pine tree) in comparison to the commercial pine oil that was used for BID-SDME method development and optimization.

Consequently, a lower plant sample amount of 0.5 g was used for further experiments. The crushed leaves were thus incubated at 55 °C in a GC-vial in a water bath and sampled using the optimised parameters. Figure 7 shows representative chromatograms of the GC-MS analysis of *T. minuta* and *P. radiata* in comparison to the commercial pine oil that was used for method development and optimisation.

Table 1 lists some of the obtained VOCs with their relative abundances (%) and their annotations according to the mass spectral library (Wiley, version 8) incorporated in the GC–MS Solutions software from Shimadzu. The threshold for positive annotation was 90% similarity match, and that coincided with the minimum abundance of ±1.5% in any one of the plant species. This was because it was observed that the match dropped to below 90% for some compounds whose relative abundances were below 1%.

**Figure 8:**
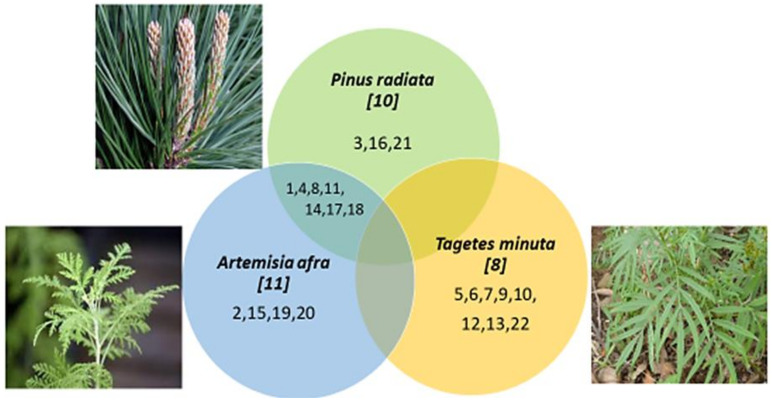
Venn diagram of shared and specific VOCs detected by GC-MS in the VOC fractions as sampled by the BID-SDME method. The number in italics indicates the total number of annotated terpenes/terpenoids. The numbers within the circles correspond to the numbered chemicals as indicated in Table 1. Corresponding structures are provided in Table S1.

For a comparative analysis, the annotated VOCs (all terpenes or terpenoids) from the three different plants were compared by means of a Venn diagram (Figure 8), indicating the distribution of the VOCs between the shared vs. exclusive sections of the diagram. Structures of the VOCs are grouped in Supplementary Table S1.

### 2.4. Some Applications of the Annotated Terpenes in the Different Test Plants

The three plant species used for the evaluation of the application of the developed methodology have different reported benefits. These plants are used locally (Lesotho, Southern Africa), and they are further reported in the literature to be useful for different purposes. For example, *P. radiata* oil is used in the manufacture of some multipurpose detergents [42,43], while *A. afra* is used for the treatment of many respiratory ailments including malaria [44], and *T. minuta* is used in pharmaceuticals, nutraceuticals, perfumery, and insecticide, just to mention a few applications [45]. A very detailed review of the medical properties of several plant-derived compounds including terpenes related to cannabis has been recently reported [46]. Of the obtained compounds, β-caryophyllene was the most abundant in *A. afra* (46%) and *P. radiata* (43%). This compound is said to be unique because it is both a terpene and a “dietary cannabinoid” [47]. It is reported to act as a local anesthetic as well as having high effectiveness in the treatment of “long lasting, debilitating pain states;” consequently, it is recommended as a substitute for the highly addictive pharmaceuticals used to treat chronic pain [48]. The same review [46] provides numerous VOCs and their pharmaceutical benefits together with the original references where these compounds have been reported for each of those functions. For examples, ocimenone, found in *T. minuta* (15%) reportedly has anti-inflammatory, antifungal and antiretroviral properties. This therefore validates the importance of this study in oil-bearing plants industries.

## 3. Materials and Methods

### 3.1. Chemicals and Reagents

All the solvents, namely methanol, dodecane and heptadecane were of HPLC grade and were obtained from Sigma-Aldrich (Merck, Johannesburg, South Africa). Commercial pine oil was obtained from Chem Cleaner, which is a local detergents manufacturer.

### 3.2. Instruments and Apparatus

The initial analyses of the VOCs were carried out using a Varian 3800 gas chromatograph (GC) equipped with a flame ionisation detector (FID) fitted with a (30 m × 1 µm × 0.53 mm film thickness) SGE-BP5 (5% phenyl—95% dimethyl-siloxane) column (SGE Analytical Science, Melbourne, Australia). A constant nitrogen gas (5.0 grade) column flow rate of 5 mL/min was maintained throughout the run. The 2 µL extract was injected into the GC with the injector set in splitless mode and the inlet temperature at 200 °C, while the detector temperature was set at 280 °C. The oven program started at 50 °C held for 2 min, then ramped at 10 °C/min to 250 °C, which was then held for 4 min, resulting in a total run time of 23 min.

For the chemical profiling and annotation of the VOCs, a Shimadzu QP 2010 GC-MS (Kyoto, Japan) fitted with a Restek Rtx-5ms (5% phenyl—95% dimethyl-polysiloxane) capillary column (Bellefonte, PA, USA), with dimensions of (30 m × 0.25 mm × 0.25μm) was used. All the GC settings were the same as in the preliminary experiments except the column flow rate that was set at 1 mL/min modified to achieve better resolution of the VOC peaks in the real sample to enable confident annotation. The thermal gradient programme began at 50 °C held for 4 min, ramped to 200 °C at a rate of 15 °C/min, and then ramped to 250 °C at a rate of 50 °C/min and was held for 3 min, achieving total run time of 20 min. The sample injection mode was splitless, with a sampling time of 2 min followed by a split ratio of 1:10 using ultra-high purity helium (Afrox, Midrand, South Africa) as the carrier gas pumped through the column at a constant flow rate of 1 mL/min. With regard to the mass spectrometer (MS) settings, the temperatures of the transfer line and ion source were at 250 °C and 200 °C, respectively, with a scanning mode used in the range of 50–500 atomic mass units (amu).

### 3.3. Extraction Method Optimisation Using Commercial Pine Oil

Individual volumes of the commercial pine oil sample were transferred into 2 mL GC vials and subjected to the SMDE extraction using a 10 µL Hamilton gastight syringe containing a certain volume of the organic solvent (the actual volume depends on the individual experiment as stated in the appropriate section). Briefly, the GC-vial cap was pierced with the syringe, and the organic solvent was pushed out slowly to be exposed in the HS volume above the aqueous suspension of the oil. The syringe was clamped on a retort stand for a set equilibration time. Thereafter, the organic droplet was retracted back into the syringe and injected into the GC. A simple water bath was set up by using a beaker filled with some water on a temperature adjustable hotplate. A mercury thermometer was used to monitor the temperature during the extraction.

Similarly, for BID-SDME, after a 2 µL volume of the organic solvent was drawn into the syringe, a corresponding volume of air was drawn in. Thereafter, the whole contents of the syringe were expunged into the HS of the aqueous solution with care not to lose the droplet. The bubbles became embedded in the droplet, forming the bubble-in-drop configuration. After the equilibration time was complete, the whole contents were retracted back into the syringe and injected into the GC.

### 3.4. Application of the Method on Real Plant Samples

Leaves from plant samples *Pinus radiata* D. Don, *Tagetes minuta* L. (wild marigold or ‘kakiebos’) and *Artemisia afra* Jacq. ex Willd (African wormwood) were harvested during the summer (October month) at the Roma University campus (Maseru in Lesotho in Southern Africa) just before the extraction to prevent any loss of VOCs that may occur during storage of the sample. Different amounts of fresh plant leaves were obtained and crushed loosely using a clean and dry pestle and mortar to break the leaves open. Crushed material (0.5 g) was weighed into 2 mL GC vials and capped with rubber/PTFE septa until further sampling. The vials were then incubated at 55 °C in the water bath for the equilibration time during the extraction.

Following the GC-MS analyses of the samples, the Wiley (Version 8) mass spectral library (www.wiley.com), incorporated in the operating GC–MS Solutions software was used to annotate the compounds.

## 4. Conclusions

This report demonstrates the applicability of the rarely reported BID-SDME method in the rapid screening of VOCs in plant samples. The optimum conditions are as follows: 1 µL of heptadecane with 0.5 µL of air bubble, 0.5 g sample in a 2 mL GC-vial incubated at 55 °C with 15 min extraction time. This approach is quite rapid with a total experimentation time of about 45 min per sample—less than 5 min grinding and weighing, 15 min of extraction/equilibration, 20 min of instrumental analyses and less than 5 min of data analysis. The 15 min extraction time is about three times faster than the SPME methods used for screening, thus making the BID-SDME method one of the most time-saving techniques for routine screening. The report further shows that the reduction in HS improves the extraction of the VOCs due to increased concentration in the HS. In application with three real plant samples, the method showed satisfactory performance. Different VOCs were obtained that were also reported elsewhere in the literature for the same plants. Clearly, the method shows promise for routine screening of the essential oils obtained from plant tissues. It can therefore be argued that this approach affords a cheaper option to a relatively more expensive SPME approach for routine screening of VOCs in the essential oil industry or for the phytoprospecting of indigenous plants with previously uncharacterised oils.

## Figures and Tables

**Table 1:** Annotation of the most abundant volatile organic compounds obtained from different plant leaves following headspace bubble-in-drop single drop microextraction (BID–SDME) sampling and gas chromatography with mass spectrometry (GC–MS) detection. Structures of the compounds are shown in Table S1.

Ret. Time (min)	#. Compound Name *	Relative Abundance (%) of Different VOCs ^$^			Presence Reported (In Similar/Different Plants)
		*P. radiata*	*T. minuta*	*A. afra*	
5.75	1. β-Ocimene (E)	3.67	-	3.68	*P. radiata, A. afra* [16,28]
6.47	2. Sabina ketone	-	-	2.08	*A. afra* [29]
6.56	3. Sabinene	2.10	-	-	*P. radiata* [30], *H. suaveolens* [31]
6.88	4. Myrcene	2.51	-	2.55	*P. radiata, A. afra* [10]
7.70	5. Limonene	-	1.53	-	*T. minuta* [32]
7.77	6. β-Ocimene (Z) ^#^	-	4.63	-	*T. minuta* [33]
8.12	7. Dihydrotagetone	-	6.97	-	*T. minuta* [34,35]
8.72	8. Terpinolene	5.85	-	5.94	*P. radiata, A. afra* [9]
8.77	9. Tagetone (E)	-	3.63	-	*T. minuta* [33]
9.45	10. Tagetone (Z)	-	15.37	-	*T. minuta* [33]
10.30	11. Terpinen-4-ol	2.00	-	1.86	*P. radiata*, *A. afra* [8]
11.26	12. Ocimenone (E)	-	15.40	-	*T. minuta* [33]
11.46	13. Ocimenone (Z)	-	29.25	-	*T. minuta* [33]
12.04	14. β-Caryophyllene (Z)	43.84	-	45.85	*P. pinaster* [36]
12.29	15. α-Bisabolene (Z)	-	-	7.4	*A. afra* [37]
12.39	16. Santolinatriene	5.99	-	-	*P. radiata* [28], *Cotula cinerea* [38]
12.78	17. Phenethyl isovalerate	9.71	-	8.70	*P. radiata*, *A. afra* [10]
12.93	18. Caryophyllene oxide	2.50	-	1.39	*P. radiata, A. afra* [10]
13.25	19. β-Elemene	-	-	1.66	*A. afra* [39]
13.68	20. Limonene oxide (E)	-	-	2.99	*A. afra* [40]
14.08	21. Caranone (Z)	7.60	-	-	*P. radiata* [28]
15.53	22. Artemisia ketone	-	6.48	-	*T. minuta* [41]
Total composition of the VOC annotated (%)		88.14	83.26	84.97	

* Annotation of the compounds was made using the Wiley Mass Spectral Library (www.Wiley.com, version 8, accessed on 15 September 2022) incorporated in the GC-MS Solutions^®^ software version 2 (Shimadzu, Kyoto, Japan), for compounds with more than 1% abundance and 90% match with the compound entries in the Wiley Library. ^$^ Relative abundance calculated as a percentage ratio of the peak area of each compound to total peak area. ^#^ It has been noted elsewhere that the *trans* isomer retains more than a *cis* isomer in a 5% phenyl column.

## Data Availability

Not applicable.

## Supplementary Materials

The following supporting information can be downloaded at: https://www.mdpi.com/article/10.3390/plants11202749/s1, Table S1: Structures of terpenes and terpenoids detected by GC-MS in the VOC fractions as samples by the BID-SDME method. The numbered chemicals correspond to that used in Table 1 in the main text. Structures were obtained from Pubchem (https://pubchem.ncbi.nlm.nih.gov, accessed on 10 October 2022).
