# Supplementary material for: Bioprospecting of Essential Oil-Bearing Plants: Rapid Screening of Volatile Organic Compounds Using Headspace Bubble-in-Drop Single-Drop Microextraction for Gas Chromatography Analysis
Source: Plants (Basel). 2022 Oct 17;11(20):2749. doi: 10.3390/plants11202749 (PMC9609334; doi:10.3390/plants11202749)
Supplement: Supplementary file 1 [file plants-11-02749-s001.zip › plants-1947792-supplementary.pdf]

Supplementary file

Bioprospecting of Essential Oil-Bearing Plants: Rapid Screening of Volatile Organic Compounds Using Headspace Bubble-in-Drop Single-Drop Microextraction for Gas Chromatography Analysis

Thabiso E. Letseka [1], Ntjana J. Sepheka [1], Ian A. Dubery [2,*] and Mosotho J. George [1,2,*]

[1] Department of Chemistry and Chemical Technology, National University of Lesotho, P.O. Box 180, Roma 100, Lesotho
[2] Department of Biochemistry, University of Johannesburg, P.O. Box 524, Auckland Park 2006, South Africa
* Correspondence: idubery@uj.ac.za (I.A.D.); jm.george@nul.ls (M.J.G.)

Table S1. Structures of terpenes and terpenoids detected by GC-MS in the VOC fractions as samples by the BID-SDME method. The numbered chemicals correspond to that used in Table 1 in the main text. Structures were obtained from Pubchem (https://pubchem.ncbi.nlm.nih.gov, accessed on 10 October 2022).

**1. , 6.** 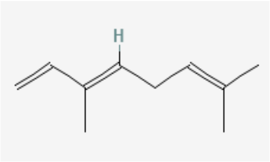	**2.** 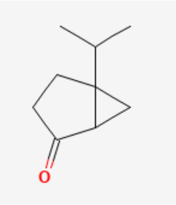	**3.** 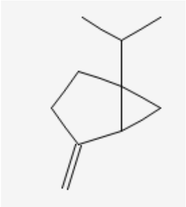	**4.** 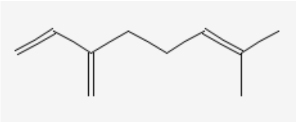	**5.** 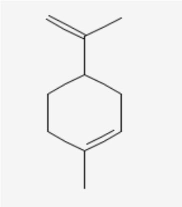
**7.** 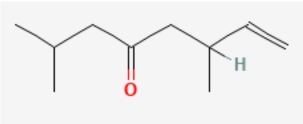	**8.** 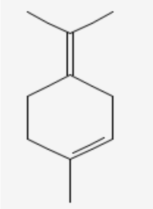	**9. , 10.** 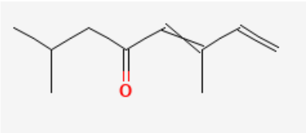	**11.** 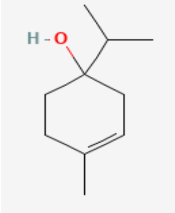	**12. , 13.** 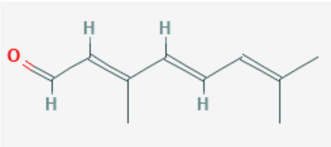
**14.** 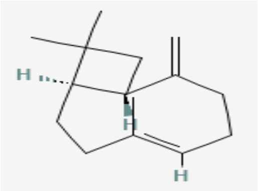	**15.** 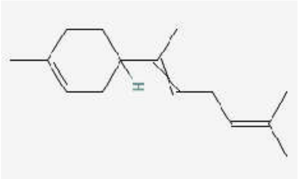	**16.** 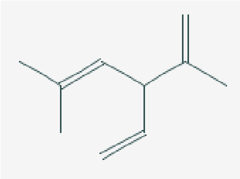	**17.** 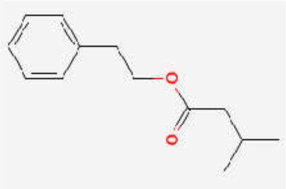	**18.** 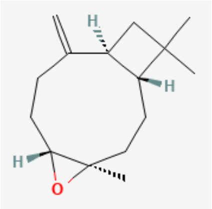
**19.** 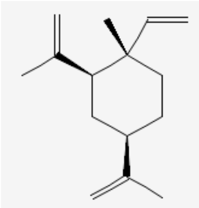	**20.** 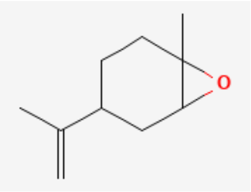	**21.** 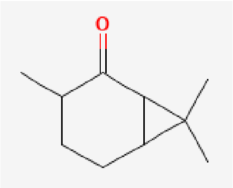	**22.** 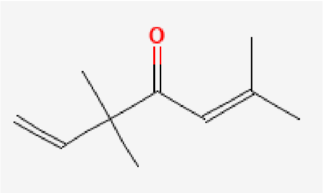	
